# Supplementary material for: The interactome of intact mitochondria by cross-linking mass spectrometry provides evidence for coexisting respiratory supercomplexes
Source: Mol Cell Proteomics. 2017 Dec 8;17(2):216–32. doi: 10.1074/mcp.RA117.000470 (PMC5795388; doi:10.1074/mcp.RA117.000470)
Supplement: Supplemental Data [file supp_RA117.000470_133922_0_supp_23978_fzffwf.zip › spectra_annotation/mito_DR_spectra_annotation/56-1-10-1-5-1.pdf]

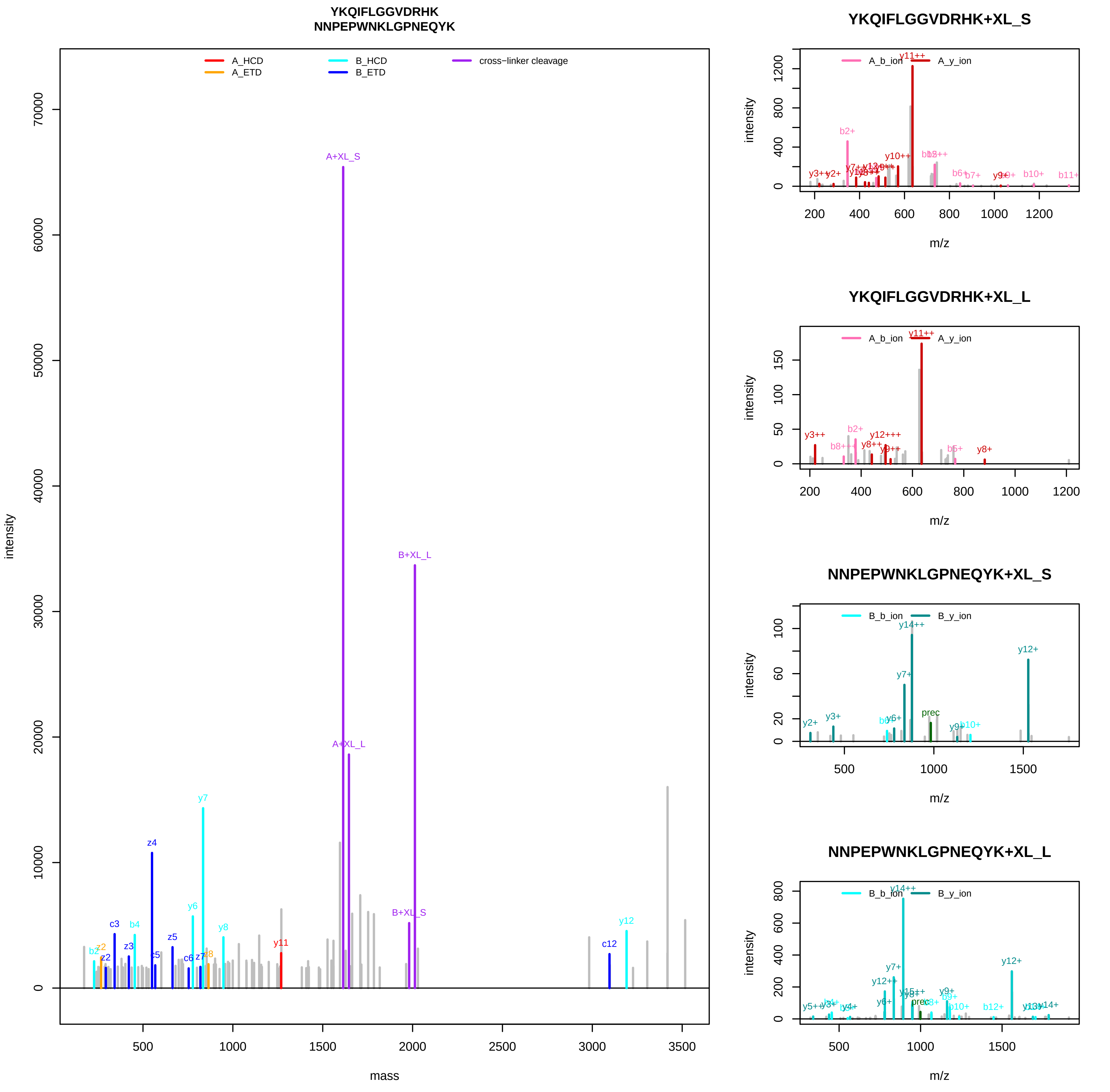

YKQIFLGGVDRHK
NNPEPWNKLGPNEQYK
A_HCD
A_ETD
B_HCD
B_ETD
cross−linker cleavage
intensity
mass
A+XL_S
A+XL_L
B+XL_L
B+XL_S
y7
y6
y8
y11
y12
c12
z4
c3
b4
z3
z5
c5
c6
z7
z8
z2
b2
YKQIFLGGVDRHK+XL_S
YKQIFLGGVDRHK+XL_L
NNPEPWNKLGPNEQYK+XL_S
NNPEPWNKLGPNEQYK+XL_L
A_b_ion
A_y_ion
B_b_ion
B_y_ion
y11++
b2+
y10++
y3++
y2+
b6+
b7+
b10+
b11+
y8++
y12+++
b8+++
b5+
y8+
y14++
y12+
y7+
y6+
y3+
y2+
prec
y9+
b10+
b12+
y13+
y14+
y5++
y15++
m/z
